# Enhancing Bone Healing with a Priming Stimulus

**DOI:** 10.3390/life16071111

**Published:** 2026-07-03

**Authors:** Michael Tanzer, Misghana Kassa, Nitin Chandra Teja Dadi, Tarek Klaylat, Rahul Gawri, Paul Martineau, Adam Hart

**Affiliations:** 1 Division of Orthopaedic Surgery, McGill University, Montreal, QC H3G 1A4, Canada; 2 Jo Miller Orthopaedic Research Laboratory, McGill University Health Centre, Montreal, QC H3G 1A4, Canada; 3 Regenerative Orthopaedics and Innovation Laboratory, McGill University Health Centre, Montreal, QC H3G 1A4, Canada

**Keywords:** priming stimulus, bone regeneration, osteoimmunology, trained immunity, bone remodeling, femoral defect

## Abstract

Bone healing is a complex regenerative process regulated by interactions between skeletal and host biologic responses, and failure of bone repair remains a major challenge in orthopedic surgery. Using a murine model, this study investigated whether a preemptive priming stimulus could enhance healing of a subsequent contralateral cortical bone defect and whether the type and timing of the stimulus influenced this response. Skeletally mature male mice were randomized into six groups (*n* = 6/group) receiving either no stimulus, a skin incision, skin and muscle incisions, or a unicortical femoral drill hole stimulus. A subcritical-sized 1 mm × 2 mm unicortical defect was subsequently created in the contralateral femur after intervals of 2, 6, or 12 weeks, depending on group allocation. Femora were harvested 8 weeks later for micro-computed tomography, histology, and immunofluorescence analyses. Mice undergoing muscle elevation 2 weeks prior to defect creation and mice receiving drill hole stimulus 12 weeks prior demonstrated the greatest degree of cortical regeneration and healing of the contralateral subcritical-sized defect, with normalized cortical thicknesses reaching 104% and 109% of adjacent native cortex, respectively. Histologic analysis confirmed restoration of mature cortical architecture in these groups. Immunofluorescence analysis demonstrated a relative shift toward an Arg1-associated reparative macrophage profile with reduced iNOS-associated inflammatory signaling, suggesting that modulation of the innate immune response contributed to the enhanced regenerative healing observed. These findings demonstrate that priming stimuli can enhance subsequent bone healing in a timing- and stimulus-dependent manner and may represent a novel strategy to optimize bone regeneration.

## 1. Introduction

Bone is a regenerative tissue that relies on complex interdependent processes to restore and maintain its physiological and biomechanical properties following injury. Bone healing is regulated by a variety of cell types and signaling pathways that together create a microenvironment capable of recruiting and activating the cells required for successful repair [1,2]. A critical component of this process is the interaction between the skeletal and immune systems, termed osteoimmunology, which describes the bidirectional communication between bone and immune cells during tissue repair and regeneration [1,2,3]. The immune microenvironment is increasingly recognized as a key determinant of both the speed and quality of bone healing [2,3].

Among immune cells, macrophages play a particularly important role in skeletal regeneration. Following injury, inflammatory macrophages contribute to debris clearance and host defense, while subsequent polarization toward reparative phenotypes promotes angiogenesis, mesenchymal stromal cell recruitment, extracellular matrix deposition, and tissue regeneration [4,5,6]. Recent studies have demonstrated that disruption of macrophage function impairs fracture healing, while modulation of macrophage activity can enhance bone regeneration [7,8,9]. Collectively, these findings suggest that the immune response is not merely a consequence of injury but an active regulator of skeletal repair.

Bone healing is integral for the success of various elective orthopedic surgical procedures such as osteotomies, limb lengthening, spinal fusions, and arthroplasty revisions [10]. Failure of bone healing results in non-union, pseudarthroses, prolonged patient recovery, and poor patient outcomes. It also represents a significant burden on the healthcare network due to direct costs for hospitalization, rehabilitation, and long-term care, as well as indirect costs relating to productivity loss [11,12,13]. In an attempt to enhance bone healing, numerous strategies have been used both intraoperatively and postoperatively. Conventional techniques include autologous and allogenic bone grafts, but they have many disadvantages, including patient morbidity, limited availability, the risk of infectious disease, and immunological rejection [14,15,16,17]. Other intraoperative bone grafting options include synthetic bone grafts such as hydroxyapatite, and growth factors/bioactive molecules such as Bone Morphogenetic Proteins (BMPs), Platelet-Derived Growth Factor-BB (PDGF-BB), and iFactor (P-15) [18,19,20,21,22]. Serious safety concerns have been reported with recombinant bone-morphogenic protein, and there is no long-term data on the safety and effectiveness of the growth factors and bioactive molecules [18,19,20,21,22]. External physical stimulation such as electric fields and ultrasound have also been used postoperatively, with variable results [23,24]. Despite these efforts to enhance bone healing, reproducible and consistent bone healing remains an elusive goal.

Preoperatively, the risk of the bone not healing can be minimized by optimizing the patient’s health. Obesity, smoking, lack of sleep, alcohol consumption, and stress have all been identified as modifiable preoperative patient risk factors that are associated with impaired bone healing, and therefore, correction of these factors prior to surgery is important [25]. Although the relationship between these factors and bone healing is multifactorial, they are all comorbidities that can lead to a disbalance in immune homeostasis [4,24,26,27]. Optimal immune function is promoted with a healthy diet and ideal body weight, while physical activity improves immunosurveillance and immunocompetence [28]. In contrast, smoking contributes to an altered immune set point [29], and alcohol consumption affects the number, survival, and function of both innate and adaptive immune cells, thereby interfering with immune responses [30]. Consumption of excessive quantities of alcohol can directly suppress a wide range of immune responses [31].

Recognition of the importance of the immune response in skeletal regeneration has led to increasing interest in immunomodulatory strategies to improve bone healing. Rather than directly targeting osteoblasts or mesenchymal progenitor cells, osteoimmunology-based therapies seek to optimize the host immune response and establish a regenerative microenvironment that supports bone formation [1,2,3]. Several studies have demonstrated that modulation of inflammatory pathways and macrophage activity can enhance skeletal regeneration. Loi et al. highlighted the critical role of controlled inflammation during fracture repair and proposed immune modulation as a therapeutic strategy to improve bone healing [32]. Chen et al. introduced the concept of osteoimmunomodulation and demonstrated that targeted regulation of immune responses could improve the regenerative performance of bone biomaterials [33]. Similarly, Spiller et al. demonstrated that modulation of macrophage polarization enhanced vascularization and tissue regeneration within bone scaffolds, while Sadtler et al. reported that regulation of adaptive immune responses promoted a pro-regenerative environment capable of improving tissue repair [34,35]. Together, these studies support the concept that manipulation of the immune response may represent a clinically relevant strategy for enhancing skeletal regeneration.

Although previous studies demonstrated that cell therapy with exogenous macrophages is beneficial to the healing of a variety of tissues, modulation of the host immune system would have the potential to be clinically feasible and provide a broadly applicable therapeutic strategy [4,36]. Previously, Ramirez-GarciaLuna et al. demonstrated that immunomodulation, through the induction of a controlled inflammatory response, promoted better bone repair [36]. In their murine model, creating a bone defect in the contralateral leg two weeks earlier resulted in faster bone remodeling and increased neo-angiogenesis in a subsequent bone defect. This enhanced bone response was characterized by changes in mast cell and macrophage populations, which resulted in more active recruitment of mesenchymal stromal cells. Based on these findings, the aim of this study was to determine whether a preemptive priming stimulus could result in increased bone healing, and how the type and the timing of the stimulus impact bone healing.

## 2. Methods

### 2.1. Study Design

**Figure 1:**
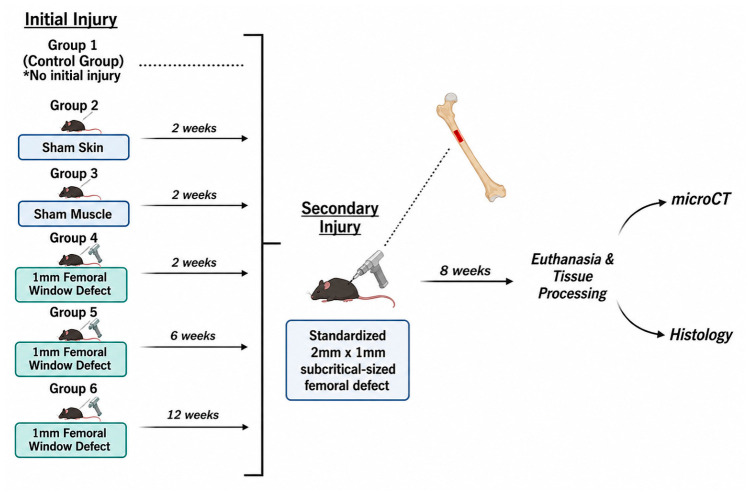
Schematic overview of the experimental design. Mice received no initial injury (control), sham skin injury, sham muscle injury, or a 1 mm femoral cortical window defect 2, 6, or 12 weeks before creation of a standardized 2 mm × 1 mm subcritical-sized femoral cortical defect (secondary injury). Eight weeks later, femora were harvested for micro-computed tomography (microCT) and histological analyses. * Control.

This study protocol was approved by the McGill University and Affiliated Hospitals Research Institutes Animal Care Committee (Protocol MUHC-7016). Skeletally mature male C57BL/6 mice (5–6 months old, Charles River Laboratories, Senneville, QC, Canada) were randomly assigned to 6 groups (*n* = 6 per group). To minimize variability in bone healing associated with hormonal fluctuations during the estrous cycle in female mice, only male mice were included in the study, thereby reducing this potential confounding factor. This study consisted of one control group and five experimental groups. The control group had no priming stimulus, while the experimental groups had either a soft tissue or bony stimulus to their right femur (Table 1). The priming stimulus was either a skin incision, skin incision and muscle elevation, or creation of a drill hole. Following the initial stimulus on their right hindlimb, all mice in the experimental cohorts had a standardized subcritical-sized defect created in their left femur. This defect was either created 2 weeks, 6 weeks, or 12 weeks after the initial stimulus (Figure 1). The 2-week interval was selected based on the findings of Ramirez-Garcia Luna et al., who demonstrated enhanced bone remodeling following a second contralateral injury created two weeks after an initial femoral defect. The 6-week interval was selected because it corresponds to the time point at which fracture healing and cortical bridging are commonly achieved in fractures. The 12-week interval was chosen to represent a more mature stage of healing and remodeling, when the acute inflammatory and reparative responses have largely resolved, and bone remodeling predominates. Together, the 2-, 6-, and 12-week intervals were selected to evaluate the influence of the priming stimulus during the inflammatory/early reparative phase, the fracture healing phase, and the late remodeling phase of skeletal repair. In the control group, there was no priming stimulus, and the mice only had the subcritical-sized defect created in their left femora. All animals were euthanized by CO_2_ asphyxiation 8 weeks following the left femoral subcritical-sized cortical defect. The healing of the cortical bone defect was analyzed qualitatively and quantitatively by micro-computed tomography (microCT) and histological analyses.

### 2.2. Surgical Procedure

The priming stimulus procedure was performed on the right limb following induction of anesthesia with 2.5% isoflurane. Under sterile conditions, a 5 mm skin incision was made over the lateral aspect of the proximal third of the femur, just distal to the greater trochanter. Mice underwent either a skin incision alone (Group 2), a skin incision with elevation of the proximal attachment of the vastus lateralis off the vastus ridge without stripping the periosteum on the femur (Group 3), or creation of a single 1 mm unicortical drill hole in the anterolateral femur (Groups 4–6).

All mice, regardless of the study group or initial intervention, subsequently underwent a second surgery involving the creation of a 1 mm × 2 mm oval-shaped unicortical femoral defect on the anterolateral aspect of the left femur.

Using the same surgical approach described for the right femur, a 1 mm drill bit was used to create a unicortical anterolateral femoral hole, followed by creation of a second adjacent hole just distal to and contiguous with the first hole. The two defects were then connected to form an oval-shaped cortical window defect. Following creation of the defect, a 1 mm × 2 mm sizing probe was used to verify defect dimensions and ensure consistency between animals. Because all procedures were performed by the same surgeon, inter-operator variability was eliminated. The wound was then copiously irrigated with normal saline to remove any bone debris in the femoral defect and the wound. The muscle, subcutaneous tissue, and skin were closed in layers. The mice started to ambulate with full weight-bearing on both legs immediately after recovery from anesthesia.

### 2.3. MicroCT Analysis

The morphology and extent of bone healing of the subcritical femoral defect of the left femur were characterized using histological techniques. Following harvest, limbs were detached through the femur and fixed in 4% paraformaldehyde for 24 h at 4 °C. Afterwards, the bone was washed in sterile phosphate-buffered saline (PBS) 3 times and then stored in PBS at 4 °C for microCT and histological analyses.

Harvested femora were scanned using a SkyScan 1172 instrument (Bruker, Kontich, Belgium) to perform micro-computed tomography (microCT) analysis at a resolution of 5 μm. Cross-sectional images were reconstructed, and three-dimensional models were generated using NRecon (v1.6.10.4, Bruker, Billerica, MA, USA) and CTvox (v2.4.0 r870, Bruker, Billerica, MA, USA). Image binarization enabled differentiation of newly formed bone at the cortical defect site from mature cortical bone. Three-dimensional inspection and differential image analysis between samples were performed using DataViewer (Bruker, Billerica, MA, USA). Quantitative analyses were conducted using CTAn (v1.16.4.1, Bruker, Billerica, MA, USA).

The cortical thickness of newly formed bone within the unicortical femoral defect was determined using the line measurement tool in CTAn. Measurements were manually obtained from sagittal cross-sectional images at the midpoint of the defect. To account for inter-animal variability in native femoral cortical thickness, cortical thickness at the defect site was normalized by dividing the thickness of the newly formed bone by the thickness of the adjacent 2 mm segment of intact cortical bone distal to the defect. Normalization of the cortical thickness eliminated the influence of variation in the normal femoral cortical thickness between the mice. In addition, using CTAn, we applied a rectangular region of interest with the dimensions of 1.0 mm length, 0.5 mm wide, and 2 mm depth, over trans axial cross-sectional images covering the length of the defect to analyze the following parameters: bone volume fraction (BV/TV%), trabecular number, trabecular separation, trabecular thickness, total porosity (%), and the number of closed pores.

### 2.4. Histological Analysis

Harvested left femora were decalcified using 10% EDTA over 3 weeks, then embedded in paraffin, sectioned, and stained using previously described protocols [36,37]. Five micron sections from the center of the unicortical defect were stained with Hematoxylin and Eosin (H&E) to evaluate the structure and morphology of the newly formed bone. High-resolution images were acquired using a Zeiss Axioskip 40 microscope (Carl Zeiss, Toronto, ON, Canada) and AxioVs40x64 (v.4.9.1.0).

### 2.5. Immunofluorescence Assay

For immunofluorescence analysis, paraffin-embedded bone sections were first deparaffinized with two 5 min washes in xylene, followed by rehydration through graded ethanol (100%, 95%, and 70%) and a rinse with distilled water. Antigen retrieval was performed by heating the slides in pH 6 citrate buffer until bubbles formed. Non-specific binding was blocked by incubating the sections in 5% BSA with 0.1% Triton-X in PBS for 30 to 60 min. Sections were then incubated overnight at 4 °C with primary antibodies against inducible nitric oxide synthase iNOS; 1:300 dilution (Invitrogen™ iNOS Recombinant Rabbit Monoclonal Antibody (HL1213), 1 mg/mL, Catalog No. PIMA546960, Fischer scientific, QC, Montreal, Canada) and Arginase-1, 1:500 dilution (Invitrogen™ Arginase 1 Polyclonal Antibody, 0.5 mg/mL, Catalog No. PIPA518392, Fischer scientific, Saint-Laurent, QC, Canada). Negative control sections were included to assess baseline fluorescence. Following three 5 min washes in 0.1% Triton X-100 in PBS, fluorophore-conjugated secondary antibodies were applied and incubated for 1 h in the dark. Secondary antibodies with higher-emission fluorophores, including Alexa Fluor 555 (Invitrogen™ Donkey anti-Goat IgG (H+L) Cross-Adsorbed Secondary Antibody, Alexa Fluor™ 555, 2 mg/mL, Catalog No. A21432, Fischer Scientific, Saint-Laurent, QC, Canada), were preferentially used to optimize signal detection. After additional washes in 0.1% Triton X-100 in PBS performed under light-protected conditions, sections were counterstained with 1 µg/mL of DAPI (Catalog No. 62248, ThermoFisher Scientific, St. Laurent, QC, Canada) in PBS for 1–10 min, rinsed with PBS, and mounted using an anti-fade mounting medium (Immumount) before coverslip application and fluorescence microscopy imaging.

#### Statistical Analysis

Normalized cortical thickness and all microCT bone parameters measured were expressed as column graphs (Mean ± SD) using the statistical program PRISM 5 v5.02. To assess the differences between the means of the bone parameters, one-way ANOVA analyses were conducted in addition to post hoc Kruskal–Wallis as a nonparametric test, and Dunn’s multiple comparisons test to reveal significance between the means of the control and experimental groups. The differences were considered significant at *p* < 0.05.

## 3. Results

**Figure 2:**
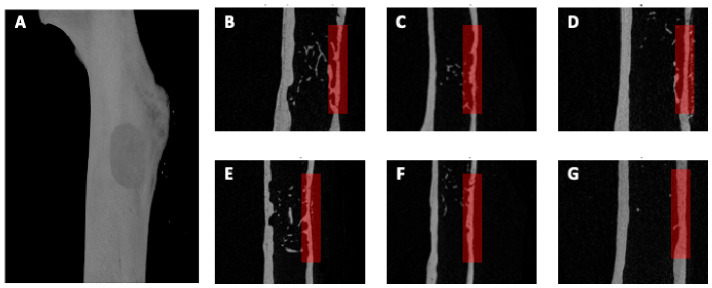
(**A**) Representative 3D microCT image of a femur from Group 6, illustrating the region of the healed oval-shaped unicortical defect. Representative 2D microCT mid-sagittal images of the left femur from (**B**) Group 1 (Control), (**C**) Group 2 (Sham Skin Group), (**D**) Group 3 (Sham Muscle Group), (**E**) Group 4 (2 weeks Delay Group), (**F**) Group 5 (6 weeks Delay Group), and (**G**) Group 6 (12 weeks Delay Group). The region in red represents that area of the secondary injury drill hole.

Differential image analysis between the 3D microCT images of each group demonstrated varying degrees of bone healing of the defect and reconstitution of the cortex in each of the groups, as well as inter-group variability (Figure 2). The partial or complete healing of all the unicortical bone defects at 8 weeks confirms that the defect was of a subcritical size and would heal.

### 3.1. MicroCT Analysis

**Figure 3:**
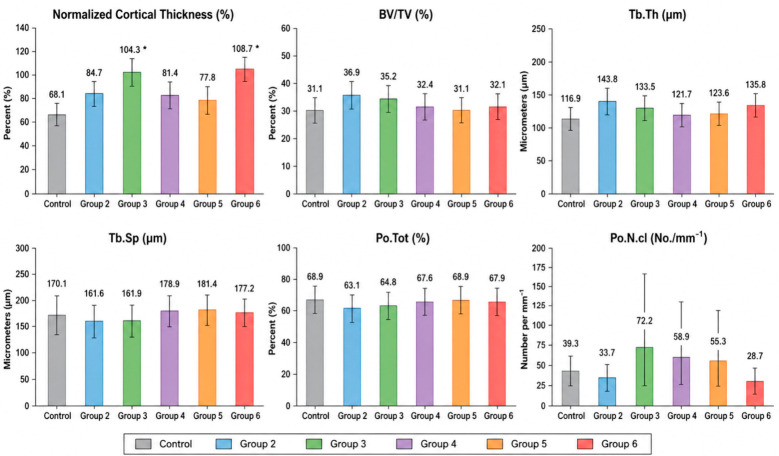
Micro-computed tomography (microCT) analyses of bone architecture and cortical healing among the experimental groups. Quantitative assessment of normalized cortical thickness, bone volume fraction (BV/TV), trabecular thickness (Tb.Th), trabecular separation (Tb.Sp), total porosity (Po.Tot), and number of closed pores (Po.N.cl) measured 8 weeks after creation of the standardized femoral cortical defect. Data are presented as mean ± standard deviation (SD). *p* < 0.05 versus the control group. Only normalized cortical thickness differed significantly between groups, with Groups 3 (sham muscle injury) and 6 (12-week femoral window defect) demonstrating greater cortical thicknesses than the controls. Abbreviations: BV/TV, bone volume/tissue volume; Tb.Th, trabecular thickness; Tb.Sp, trabecular separation; Po.Tot, total porosity; Po.N.cl, number of closed pores. * Significantly greater than Control Group.

Quantification of cortical thickness, normalized to the distal and adjacent normal bone, found that the control group, with no priming stimulus, had incomplete reconstitution of the cortical defect, only reaching a mean of 68% of its normal thickness (Table 2, Figure 3). At 8 weeks following the unicortical defect surgery, there was complete reestablishment of cortical thickness only in Groups 3 and 6, with the normalized thickness being 104% and 109%, respectively (Table 2, Figure 3). There was no difference in the normalized cortical thickness between Groups 3 and 6 (*p* < 0.05), but in both groups, cortical thickness was significantly greater than that in the control group (Group 3: *p* = 0.0042, Group 6: *p* = 0.0011) In the other groups, the mean normalized cortical thickness varied between 78 and 85%, but this was not significantly different than the controls (Table 2, Figure 3).

The mean bone volume (BV/TV) within the cortical defect region and trabecular thickness were higher in Groups 3 and 6 compared to the control; however, in all experimental groups (including 3 and 6), this was not statistically significantly different than in the control group (Table 2, Figure 3). No significant differences were observed between the control and experimental groups for any of the other assessed bone microarchitectural parameters (Table 2, Figure 3).

### 3.2. Histological Analysis

**Figure 4:**
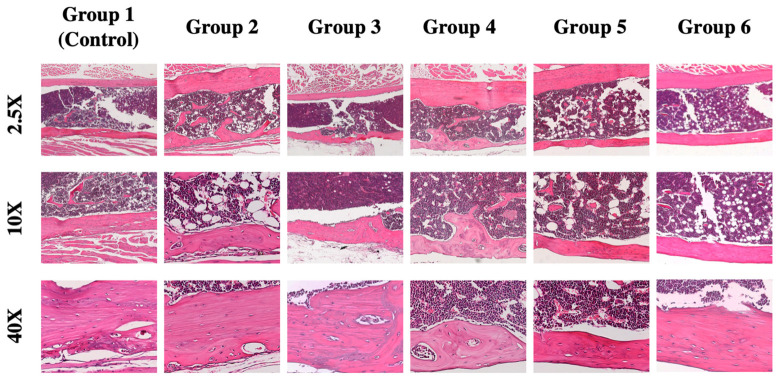
Representative sections through the middle of the defect in left femurs at 2.5×, 10×, and 40× magnification from the following groups: Group 1 (control; no priming stimulus), Group 2 (sham skin priming), Group 3 (sham muscle priming), Group 4 (femoral drill-hole priming with a 2-week delay), Group 5 (femoral drill-hole priming with a 6-week delay), and Group 6 (femoral drill-hole priming with a 12-week delay). Group 2 demonstrated greater filling of the defect region than the control group, although areas of residual porosity and cortical irregularity remained visible. Group 3 demonstrated a more continuous cortical contour across the defect with less visible porosity and a thicker regenerated cortex than the control. Groups 4 and 5 demonstrated bone formation throughout the defect region but continued to exhibit cortical irregularity and residual porous spaces within the regenerated cortex. The appearance of the regenerated cortex in these groups was similar to the control group and consistent with the absence of a significant difference in normalized cortical thickness compared with controls. Group 6 demonstrated the most complete restoration of cortical continuity across the defect. The regenerated cortex appeared thicker and more uniform than in the control group, with less visible porosity within the defect region.

Histological examination demonstrated cortical bone formation within the defect region in all groups (Figure 4). The control group showed incomplete restoration of the cortical defect, with persistent cortical irregularity, residual porous regions within the defect, and areas of newly formed bone interspersed with the remaining defect space.

Overall, the histological findings paralleled the microCT results (Table 2, Figure 3), with Groups 3 and 6 demonstrating the most complete restoration of cortical morphology, whereas the remaining groups exhibited varying degrees of residual porosity, cortical irregularity, and incomplete cortical reconstitution.

### 3.3. Immunofluorescence Assay

**Figure 5:**
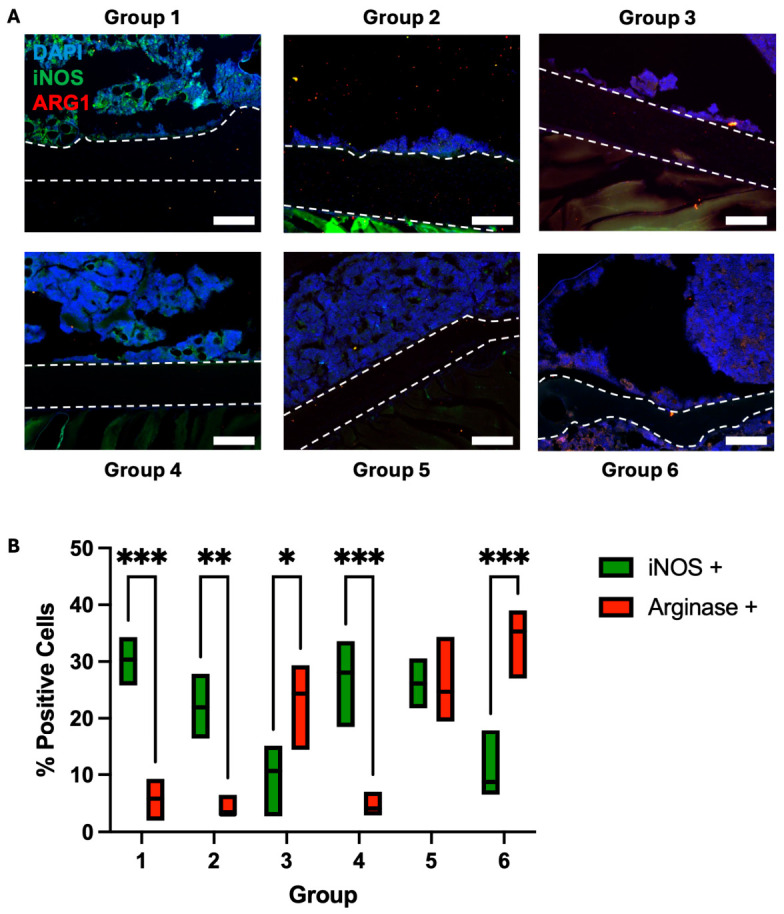
Immunofluorescence assessment of macrophage polarization within the healing cortical defect. (**A**) Representative Immunofluorescence images for each group. Dashed lines delineate cortical bone. DAPI (blue) was used to stain the cell nuclei. iNOS-positive and arginase 1 (ARG1)-positive cells are stained in green and red, respectively. Scale bar = 250 µm. (**B**) Quantification of iNOS-positive versus arginase-positive cells in each group. Quantitative data are presented as the median and distribution for each group. *n* = 6; * *p*-value ≤ 0.05, ** *p*-value ≤ 0.01, and *** *p*-value ≤ 0.001.

**Figure 6:**
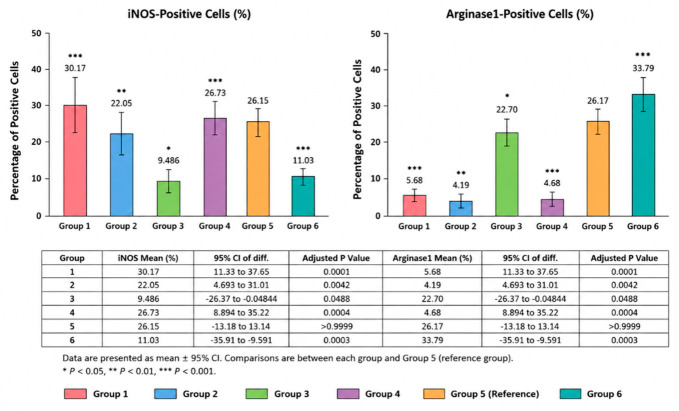
Immunofluorescence quantification of iNOS-positive and Arginase1-positive cells across experimental groups. Quantification of the percentage of iNOS-positive (pro-inflammatory) and Arginase1-positive (pro-reparative) cells within the defect region 8 weeks after creation of the standardized femoral cortical defect. Group 5 served as the reference group for all statistical comparisons. Bar graphs present mean ± 95% confidence interval (CI), and the accompanying table summarizes mean values, 95% CIs of the mean differences, and adjusted *p*-values for pairwise comparisons with the reference group. Significant differences are indicated as *p* < 0.05 (*), *p* < 0.01 (**), and *p* < 0.001 (***).

Immunofluorescence analysis was performed to characterize the macrophage polarization state across all six groups. Representative images are shown in Figure 5A, and quantitative analysis is presented in Figure 5B. Figure 5A shows representative immunofluorescence images for each of the six groups. Sections were co-labeled with antibodies against iNOS (green), a marker associated with classically activated, pro-inflammatory M1-like macrophages, and Arg1 (red), a marker associated with alternatively activated, reparative M2-like macrophages, with DAPI nuclear counterstain (blue) used to identify all cells. Across Groups 1–6, the percentage of Arg1-positive and iNOS-positive cells varied markedly between conditions (Figure 6). In Groups 1, 2, and 4 (Figure 5A), the predominant signal is green (iNOS), with comparatively sparse red (Arg1) labeling, indicating significantly higher percentages of iNOS-positive cells than Arg1-positive cells. This suggests predominance of the iNOS-associated population in these groups (*p* = 0.0001, *p* = 0.0042, *p* = 0.0004, respectively), as shown in Figure 5B. In Group 3, this pattern shifted, with Arg1-positive cells exceeding iNOS-positive cells, although the difference was smaller (mean difference −13.21, *p* = 0.0488, Figure 5B and Figure 6). Group 5 displays co-labeling of both markers present in comparable proportions, thus showing no clear dominance of either marker (*p* > 0.9999, Figure 5B). Group 6 demonstrated the opposite pattern to Groups 1, 2, and 4, with a significantly higher proportion of Arg1-positive cells and reduced iNOS-positive cells (*p* = 0.0003, Figure 5B). Overall, the data indicate that Groups 1, 2, and 4 are biased toward an iNOS-associated profile, Group 3 shows an intermediate shift toward Arg1, Group 5 appears mixed, and Group 6 is the most strongly shifted toward an Arg1-associated profile (Figure 5).

## 4. Discussion

This study investigated various priming stimuli to enhance and optimize bone healing of a subsequent contralateral, subcritical, femoral cortical defect in a murine model. Compared to the control group that had no prior stimulus, the groups that had either exposure and elevation of the overlying femoral musculature 2 weeks prior to the contralateral femoral defect, and those that had a 1 mm drill hole stimulus 12 weeks prior to their contralateral femoral cortical defect, demonstrated complete bone healing and restoration of the femoral cortical bone 8 weeks later. MicroCT and histologic analyses demonstrated that the defects in these groups healed with restoration of normal cortical bone architecture, achieving cortical thicknesses of 104% and 109% relative to the adjacent native cortex, respectively. The healed cortical thickness in both groups was significantly greater than that observed in the control group, although no significant difference was identified between the two priming stimuli. In contrast, all the other priming stimuli resulted in incomplete healing of the defect, with bone formation not significantly different than the controls. This study corroborates the findings of a previous investigation that utilized the same animal model and priming stimulus (Group 4) [27]. A priming stimulus consisting of a 1 mm femoral bone hole, followed 2 weeks later by the creation of a 1 mm × 2 mm oval-shaped unicortical defect in the contralateral anterolateral femur, did not result in greater bone formation compared to the control animals with no stimulus [27]. However, the previous study demonstrated that this priming stimulus resulted in a transient increase in the inflammatory response that promoted faster bone remodeling and neo-angiogenesis. This priming stimulus was characterized by changes in mast cell and macrophage content (macrophage priming) that translated into more active recruitment of mesenchymal stromal cells. There was an increase in both anabolic and catabolic macrophage markers after 2 weeks and then a reversal after 8 weeks. It is this immunologic process that we postulate is responsible for the findings in this study. After exposure to certain stimuli, innate immune cells, such as macrophages, can adjust their response to subsequent insults, resulting in an enhanced response. Immune priming is the process whereby an initial stimulus changes the functional state of the innate immune cells, and they do not return to homeostasis before a secondary stimulus, which results in an enhanced response of the primed cells [30]. Whereas trained immunity is the process where innate immune cells are exposed to an initial stimulus resulting in persistent epigenetic changes, and re-stimulation of that innate immune cell results in an enhanced response compared with the initial stimulation [30]. In the context of this study, we postulate that the osteoimmunology response was dependent on the stimulus and the duration between the initial and secondary stimuli. In the Sham Muscle Group (Group 3), exposure and elevation of the overlying femoral musculature was performed 2 weeks prior to the contralateral femoral defect. After the acute muscle injury, pro-inflammatory M1 macrophages arrive first to clear the damaged tissue and undergo a phenotypic transition to support the growth of new tissue [21,31]. Before the pro-inflammatory priming stimulus subsided and the functional state of these cells returned to their pre-stimulation levels, the second stimulus resulted in the enhancement of the inflammatory response. These pro-inflammatory M1 macrophages are sequentially replaced by healing M2 macrophages that sustain tissue repair and regeneration [31,32].

The immunofluorescence findings closely paralleled both the microCT and histological outcomes. Groups 3 and 6, which demonstrated the greatest cortical regeneration and most complete restoration of cortical morphology, also exhibited the most favorable Arg1/iNOS profiles. Because elevated iNOS expression is associated with a pro-inflammatory M1-like macrophage phenotype, whereas Arg1 expression is associated with a reparative M2-like phenotype, these findings suggest that enhanced healing was accompanied by a shift toward a more regenerative immune environment [35,36]. Although causality cannot be established from the present study, the concordance between macrophage polarization and structural healing outcomes supports the hypothesis that modulation of innate immune responses contributed to the enhanced bone regeneration observed.

We theorize that the extended time between stimuli in Group 6 resulted in trained immunity of the inflammatory macrophages. It is probable that the epigenetic changes in the macrophages that occurred over the 12-week time frame between stimuli can be responsible for the enhanced bone healing after re-stimulation.

The relationship between bone and the immune system is bidirectional and determines the ability for bone tissue to regenerate and remodel [33]. However, if immune cells are facilitating chronic inflammation, this can lead to the suppression of factors and a microenvironment conducive to quality bone regeneration [34]. We postulate that this was the case for Groups 4 and 5, whereby the time elapsed between stimuli was not long enough for the level of injury induced, suppressing the anabolic function of the acute inflammation needed for bone regeneration. For Group 2, the skin incision alone (Group 2) appears to be an insufficient priming stimulus to promote an enhanced response.

Previously, our collaborative group described a double-fracture murine model that showed evidence of a controlled increase in the inflammatory state of healing a fracture due to a previously sustained injury within a short period, which resulted in a higher rate of bone remodeling and increased neo-angiogenesis [27]. The double-fracture group, with one femoral defect 2 weeks prior to another femoral defect on the contralateral limb, revealed a significant increase in the number of macrophages and alternatively activated M2 macrophages. Quantitative analyses of the double-fracture group revealed an increase in both anabolic and catabolic macrophage markers 2 weeks post-operation and then a reversal after 8 weeks. The early increase in macrophages, in this context, supports the conjecture that it plays an important immunoregulatory function in tissue repair and regeneration properties in the enhanced phenomenon.

These findings suggest that macrophage-associated polarization is group-dependent during fracture repair, with Group 6 showing the clearest pro-reparative Arg1-dominant pattern, whereas Groups 1, 2, and 4 retain a more pro-inflammatory iNOS-dominant profile. Group 3 may represent a transitional state, and Group 5 may reflect the coexistence of both phenotypes. These findings suggest a group-dependent shift in the fracture microenvironment from a predominantly pro-inflammatory (iNOS-associated) profile toward a more pro-reparative (Arg1-associated) phenotype. However, in the absence of a pan-macrophage marker, these results should be interpreted cautiously, as Arg1 and iNOS expression cannot be definitively attributed to macrophages or used to assess total macrophage infiltration.

A limitation of this study is that inducing a priming stimulus by producing a bone defect is not clinically feasible due to safety concerns. Other limitations include bone formation data that were calculated from microCT data and revealed no significance, which is why the cortical thickness was measured as a relative value to normal and adjacent bone. This measurement was standardized but is not a standard measure on the CT scan software used to calculate other bone parameter measurements. Future measurements to assess the quality of healed bone are bone mineral density from microCT data and mechanical testing to observe the effects of priming on restoring the physiological and biomechanical properties of healing bone. Although significant differences were detected for normalized cortical thickness, this study may have been underpowered to detect smaller differences in several of the secondary microCT parameters because each experimental group contained only six animals. While BV/TV and trabecular measurements were generally higher in Groups 3 and 6 than in the control group, these differences did not reach statistical significance. Therefore, the absence of significant differences in these secondary outcomes should be interpreted with caution, as larger studies may be required to determine whether these trends reflect meaningful biological effects. Future studies should aim to expand the scope of experimental groups so that the effects of priming vs. training can be studied per type of priming and elucidate what time range is best for an enhanced immune response. In addition, bone samples should be sent for proteomic analyses to identify the epigenetic modifications so that immunomodulatory effects can be exploited. The evidence supporting trained immunity remains indirect since no direct assessment of trained immunity, epigenetic modifications, chromatin remodeling, transcriptional profiling, or memory-associated innate immune responses was performed. Finally, the translational implications of these findings should be interpreted within the limitations of a murine model. Differences in skeletal biology, immune function, and healing kinetics may influence the applicability of these results to humans. Nevertheless, the ability of a prior musculoskeletal stimulus to induce a prolonged pro-regenerative immune response raises the possibility that immune conditioning strategies could be used to enhance bone healing in clinical practice. Future studies should investigate whether targeted immunomodulatory interventions can reproduce these effects in larger animal models and ultimately in patients.

## 5. Conclusions

This murine study provides preliminary evidence that preemptive musculoskeletal stimulus can enhance subsequent bone healing in a stimulus- and time-dependent manner. The greatest regenerative response was observed in mice that underwent muscle elevation two weeks before defect creation and those that received a femoral drill hole stimulus twelve weeks earlier, restoring normalized cortical thickness to 104% and 109% of the adjacent native cortex, respectively. Histological findings paralleled the microCT results, while immunofluorescence analysis demonstrated a shift toward an Arg1-associated reparative macrophage phenotype and reduced iNOS-associated inflammatory signaling, supporting an osteoimmunologic basis for the enhanced healing response.

The results of this study provide preliminary evidence that a priming stimulus via controlled injury to muscle overlaying femoral bone could be used to enhance bone healing of a subsequent bone injury. This technique to optimize bone regeneration and remodeling is favorable because it is minimally invasive, making it more feasible in a clinical setting and can serve as an important protective strategy that prevents poor bone healing outcomes.

Although limited by its murine model, small sample size, and lack of biomechanical testing, this study suggests that the host immune system can be conditioned to respond more effectively to subsequent skeletal injury. These findings advance our understanding of the relationship between immunity and skeletal regeneration and raise the possibility that immune conditioning before injury or surgery may represent a novel strategy to enhance bone healing. Future studies should define the molecular and epigenetic mechanisms underlying this response, identify the optimal priming stimulus and timing, and evaluate clinically applicable immunomodulatory approaches for improving orthopedic healing.

## Figures and Tables

**Table 1:** The six experimental groups differentiated by the type and timing of the priming stimulus.

	Priming Stimulus on Right Proximal Femur	Subcritical Femoral Defect in Left Proximal Femur Timing	Right and Left Femoral Harvesting
Group 1 (Control Group)	None	Initially	8 weeks following left femoral defect
Group 2 (Sham Skin Group)	5 mm skin incision	2 weeks following priming stimulus	8 weeks following left femoral defect
Group 3 (Sham Muscle Group)	5 mm skin and muscle incision	2 weeks following priming stimulus	8 weeks following left femoral defect
Group 4 (2 weeks Delay Group)	1 mm femoral drill hole	2 weeks following priming stimulus	8 weeks following left femoral defect
Group 5 (6 weeks Delay Group)	1 mm femoral drill hole	6 weeks following priming stimulus	8 weeks following left femoral defect
Group 6 (12 weeks Delay Group)	1 mm femoral drill hole	12 weeks following priming stimulus	8 weeks following left femoral defect

**Table 2:** microCT analysis of bone parameters with the mean and standard deviation: BV/TV, Bone Volume/Tissue Volume; Tb.Th, Trabecular Thickness; Tb.Sp, Trabecular Separation; Po(tot), Total Porosity; Po.N.cl, Number of Closed Pores and Normalized Cortical Thickness.

	Control	Group 2	Group 3	Group 4	Group 5	Group 6
Normalized Cortical ±Thickness (%)	68.1 ± 13.7	84.7 ± 18.5	104.3 ± 15.9 *	81.4 ± 11.1	77.8 ± 22.5	108.7 ± 6.1 *
BV/TV (%)	31.1 ± 4.9	36.9 ± 5.7	35.2 ± 2.6	32.4 ± 4.3	31.1 ± 3.4	32.1 ± 3.9
Tb.Th (µm)	116.9 ± 17.9	143.8 ± 23.5	133.5 ± 12.5	121.7 ± 12.4	123.6 ± 13.8	135.8 ± 17.1
Tb.Sp (µm)	170.1 ± 15.9	161.6 ± 17.7	161.9 ± 11.9	178.9 ± 30.9	181.4 ± 25.3	177.2 ± 17.1
Po.Tot (%)	68.9 ± 4.9	63.1 ± 5.7	64.8 ± 2.6	67.6 ± 4.3	68.9 ± 3.4	67.9 ± 3.9
Po.N.cl (No./mm^−1^)	39.3 ± 36.7	33.7 ± 29.9	72.2 ± 85.5	58.9 ± 92.7	55.3 ± 78.2	28.7 ± 10.1

* Indicates value is significantly different than the control.

## Data Availability

Dataset available on request from the authors.
